# Induction of Cytopathogenicity in Human Glioblastoma Cells by Chikungunya Virus

**DOI:** 10.1371/journal.pone.0075854

**Published:** 2013-09-25

**Authors:** Rachy Abraham, Prashant Mudaliar, Aiswaria Padmanabhan, Easwaran Sreekumar

**Affiliations:** Viral Disease Biology Program, Rajiv Gandhi Centre for Biotechnology (RGCB), Thiruvananthapuram, Kerala, India; Agency for Science, Technology and Research - Singapore Immunology Network, Singapore

## Abstract

Chikungunya virus (CHIKV), an arthritogenic old-world alphavirus, has been implicated in the central nervous system (CNS) infection in infants and elderly patients. Astrocytes are the major immune cells of the brain parenchyma that mediate inflammation. In the present study we found that a local isolate of CHIKV infect and activate U-87 MG cells, a glioblastoma cell line of human astrocyte origin. The infection kinetics were similar in infected U-87 MG cells and the human embryo kidney (HEK293) cells as indicated by immunofluorescence and plaque assays, 24h post-infection (p.i.). In infected U-87 MG cells, apoptosis was detectable from 48h p.i. evidenced by DNA fragmentation, PARP cleavage, loss of mitochondrial membrane potential, nuclear condensation and visible cytopathic effects in a dose and time-dependent manner. XBP1 mRNA splicing and eIF2α phosphorylation studies indicated the occurrence of endoplasmic reticulum stress in infected cells. In U-87 MG cells stably expressing a green fluorescent protein-tagged light chain-3 (GFP-LC3) protein, CHIKV infection showed increased autophagy response. The infection led to an enhanced expression of the mRNA transcripts of the pro-inflammatory cytokines IL-1β, TNF-α, IL-6 and CXCL9 within 24h p.i. Significant up-regulation of the proteins of RIG-I like receptor (RLR) pathway, such as RIG-I and TRAF-6, was observed indicating the activation of the cytoplasmic-cellular innate immune response. The overall results show that the U-87 MG cell line is a potential *in vitro* model for in depth study of these molecular pathways in response to CHIKV infection. The responses in these cells of CNS origin, which are inherently defective in Type I interferon response, could be analogous to that occurring in infants and very old patients who also have a compromised interferon-response. The results also point to the intriguing possibility of using this virus for studies to develop oncolytic virus therapy approaches against glioblastoma, a highly aggressive malignancy.

## Introduction

Chikungunya virus (CHIKV) is an arthritogenic old-world alphavirus that has re-emerged exhibiting neurotropism [1]. CNS complications such as severe encephalitis, meningoencephalitis, peripheral neuropathies, encephalopathy, cerebral haemorrhage, as well as deaths among newborns, infants and elderly patients were evidenced in the recent outbreaks [2,3,4]. In contrast to the true neurotropic virus infections, the molecular mechanism of CHIKV neurotropism is still not clearly defined. However, the property is thought to have emerged in conjunction with the adaptive evolutionary changes in the viral genome [5] as the newer strains of CHIKV that led to complications harboured several novel genetic changes compared to the classical strains of the virus which usually cause an acute febrile illness with arthralgia and myalgia [6]. The defining role of the mutations resulting from these genetic changes in neurovirulence or neuroinvasiveness has not been explored so far even though some of them are shown to enhance mosquito adaptability [7].

CHIKV has been shown to infect a large variety of cells of different lineages (Table 1). In view of this broad *in vitro* cell tropism exhibited by CHIKV in a dose-dependent manner, a hypothesis could be that the neurovirulence is due to a spill-over infection as generally observed in other arbovirus CNS infections [8]. Thus, the viremia caused by newer CHIKV strains in patients reaches beyond a threshold level enabling the virus to cross the blood-brain barrier establishing the brain infection. Supporting this assumption, extremely high viremia (to the order of 10^8^ pfu/ml) has been reported in chikungunya patients with complications during out-breaks occurred in Ré Union island [1]. The viremia would be further augmented both in the periphery as well as in the brain parenchyma by a poor Type I interferon (IFN) response in infants and very old patients [9,10]. Also, in young age animal models, CHIKV that is introduced directly into brain establishes infection and exhibits neurovirulence by infecting stromal cells of the central nervous system and inducing severe vacuolization of choroid plexus epithelial cells and ependymal cells [11]. These strains also cause direct infection of mouse astrocytes [1] in culture indicating the permissibility of CNS cells to infection.

**Table 1:** Human cell-based in vitro models reported so far for CHIKV infection studies.

**Sl No**	**Cell Name**	**Source/Cell type**	**Susceptibilityto infection**	**Visible Cytopathic effect**	**Innate immune response**	**Apoptosis**	**Autophagy**	**ER Stress**	**Pro-inflammatorycytokine response**	**References**
**Primary Cells of Human Origin**										
1	CD14^+^ monocytes		+		+				+	[53,54]
2	Activated CD4+ T cells		-							[55]
3	CD19^+^ B cells		+		+					[54]
4	Monocyte-derived macrophages		+							[55]
5	Monocyte-derived dendritic cells		-							[55]
6	Peripheral blood mononuclear cells		-							[55]
7	Muscle cells		+	+						[56,57]
8	Differentiated myotube		-							[56]
9	Osteoblasts		+						+	[58]
**Cell lines of Human Origin**										
1	A549	Alveolar basal epithelial cells	-	-						[55]
2	B-420	EBV-transformed B cells	±							[55]
3	BEAS-2B	Bronchial epithelial	+++	+	+					[55,59]
4	CHME-5	Foetal Microglial	+			+				[25,60,61]
5	HEL	Embryonic lung	+	+						[62]
6	HF	Foreskin fibroblast	+		+		+			[38,49,50]
7	hCMEC/D3	Brain endothelial	-	-						[55]
8	HeLa	Cervical epithelial	+++	+		+	+	+		[25,28,38,55,63,64]
9	HEK 293, HEK293T	Embryonic kidney	+++	+		+	+	+		[25,38,39,54,55,63,64]
10	HepG2	Hepatocellular	+			+				[25]
11	HS 633T	Fibrosarcoma	++		+					[65]
12	Hs 789.Sk	Skin fibroblast	++	+						[55]
13	HT-1080	Fibrosarcoma	+		+					[65]
14	HSMM	Skeletal muscle myoblasts	++							[66]
15	Jurkat	CD4+ T lymphoid cells	±							[55,64]
16	K562	Erythromyeloblastoid Leukemia	±							[64]
17	LHCN-M2	Human Skeletal Myoblast	+							[57]
18	MRC-5, Resting MRC5	Lung fibroblast	+++	+	+	+		+		[25,50,55,64]
19	RD	Muscle rhabdomyosarcoma	+++							[67,68]
20	SH-SY5Y	Neuroblastoma	+++	+		+			+	[63,69]
21	SW-982	Synovial sarcoma	-			-				[25]
22	THP-1	Acute monocytic Leukemia	±							[55,64]
23	TrHBMEC	Bone marrow endothelial	+	+						[55]
24	U937	Leukemic monocytic lymphoma	±			-				[25,55]
25	U-87 MG	Glioblastoma (Astrocytic)	+++	+	+	+	+	+	+	Present Study

‘+ ’, indicates a positive response and ‘- ’ indicates a negative response. ‘±’ indicates a poor response. Blank cells indicate that the mentioned cellular responses were not addressed in the respective studies.

Inflammation is the key event that results in tissue damage and pathology causing long-term consequences in viral infections of the CNS [12]. The microglial cells and astrocytes within the CNS, of which the astrocytes form as much as half the mass of brain cells, constitute the major immune cells of the brain parenchyma engaged in neuroinflammation [13]. Activation of these glial cells during infection results in alterations in their physiology by endoplasmic-reticulum stress, increased production of pro-inflammatory mediators like cytokines or chemokines, oxidative stress due to overproduction of reactive oxygen and /or nitrogen species and death of the infected cells by apoptosis or necrosis [14]. Previous studies have shown that many viral infections lead to glial cell activation, for e.g. Theilers virus [15], Measles virus [16] and Newcastle disease virus [17], and some specifically cause astrocyte activation, for e.g. Sindbis virus [18] and West Nile Virus [19].

Since poor or lack of Type I IFN-response remains a critical element in neurovirulence/ neuroinflammation by CHIKV, availability of a suitable cellular model, especially of glial cell origin, to study the molecular inflammatory responses during infection under IFN-deficient conditions would be highly useful. Accordingly, to establish such a model we used U-87 MG cells, an IFN-response deficient glioblastoma cell line of astrocyte origin [20,21]. Apart from studying the primary susceptibility of these cells to a CHIKV strain causing classical chikungunya fever, we also looked into the ability of the virus to cause ER stress, autophagy, apoptosis and modulation of pro-inflammatory cytokine responses in these cells.

## Materials and Methods

### Cell culture and virus stock

U-87 MG (ATCC HTB-14), a cell line originated from a human glioblastoma multiforme, and HEK293 (Human embryo kidney cells) were obtained from American Type Culture collection (ATCC). Vero cell line (African green monkey kidney cells) was obtained from the cell repository of National Centre for Cell Sciences, Pune, India. These cells were grown in DMEM (Dulbecco’s modified Eagle’s medium) supplemented with 10% heat inactivated foetal bovine serum (FBS) and 1× antibiotic-antimycotic mixture (all from Sigma). Cultures were incubated at 37°C in a humidified atmosphere containing 5% CO_2_. Characterization of the RGCB355/KL08 CHIKV strain (GenBank Accession No. GQ428214) by whole genome sequencing has been previously described [22]. The virus was isolated by three serial passages in Vero cells prior to use for infecting U-87 MG cells. For virus quantification by plaque assay, confluent monolayer cultures of Vero cells were incubated with serial dilutions of viral stock for 2h, and then overlaid with 1.5% carboxymethyl cellulose in DMEM with 2% FBS and incubated at 37°C for 48h. The cells were fixed with 30% formalin in phosphate buffered saline (PBS; pH 7.4), stained with 0.05% crystal violet and the number of plaques was counted to calculate the virus titre.

### Infection of U-87 MG cells and HEK293 cells

Monolayer cultures of U-87 MG and HEK293 cells at 70-80% confluency was infected with RGCB355/KL08 in DMEM with 2% FBS at different multiplicity of infection (MOI) and incubated for 2h at 37°C. After giving a PBS wash to remove the un-adsorbed virus, the cells were maintained in DMEM with 2% FBS at 37°C for the required time points. For time kinetic analysis of virion production, the supernatants from infected cells were collected and ten-fold serial dilutions were processed for plaque assay as described above. At the end of the incubation period, cells were collected in Trizol (Invitrogen) for mRNA studies or in phospho lysis buffer for protein studies.

### Immunofluorescence staining

Cells were grown on glass cover slips and infected at the respective MOI. At different times post infection, cells were fixed using 4% paraformaldehyde in PBS for 15 min at 4°C and washed three times with PBS. The cells were permeabilized with 0.2% Triton X-100 for 10 min at room temperature, washed with PBS and blocked by incubating in PBS containing 5% normal goat serum for 1h at 37°C. After washing with PBS, cells were incubated in 1:10 dilution of primary antibody [in-house rabbit anti-CHIKV polyclonal serum against recombinant E1 protein] for 2h. Subsequent to three times washing with PBS, cells were incubated with 1:200 dilution of mouse anti-rabbit IgG FITC secondary antibody (Sigma). Nucleus morphology was revealed by DAPI staining (final concentration: 1µg/ml). Cover slips were mounted on glass slides and fluorescence was observed using an inverted fluorescent microscope (Nikon Eclipse Ti-S). Images were captured with a LucaR (Andor) EMCCD camera using NIS elements software under identical exposure and gain settings for the infected cells as well as the controls. Cells treated only with the secondary antibody served as background fluorescent staining control in the experiments.

### Measurement of mitochondrial membrane potential (MMP) and nuclear condensation

JC-1 powder (Molecular Probes) was dissolved in dimethyl sulfoxide at a concentration of 1 mg/ml, aliquoted, and stored frozen at -20°C. 70-80 percentage points confluent U-87-MG cells in a 24-well plate was infected or mock infected at different MOI. At various times post-infection, medium was removed and 1 ml fresh DMEM medium containing JC-1 (100 ng/ml) was added to each well. The plates were further incubated in the dark for 1 h at 37°C before visualization. Hoechst 33342 Trihydrochloride Trihydrate (Invitrogen) was dissolved in PBS and 100µl of 5µg/ml stock was added to the cells and incubated for 20 minutes at 37°C in the dark before visualization. Images were captured as described above.

### DNA fragmentation analysis

Infected cells were pelleted and lysed with the detergent buffer (10mM Tris pH 7.4, 5mM EDTA, 0.2% Triton), vortexed and kept in ice for 30 minutes. Cell debris was removed by centrifugation at 20,000 × g for 30 min; 50µl ice cold 5M NaCl was added to the supernatant and mixed well. The DNA was precipitated by addition of 150µl of 3M Sodium acetate and 600µl of 100% ethanol and incubation for one hour at -80°C followed by centrifugation at 20,000 × g for 15min at 4°C. The pellet was dissolved in extraction buffer (10mM Tris,5mM EDTA) and treated with 2µl of 10mg/ml RNase A for 5 hours at 37°C. 25µl of 20mg/ml Proteinase K and 40µl buffer (100mM Tris,pH 8, 100mM EDTA,250mMNaCl) was added and the samples were incubated overnight at 65°C. The DNA was extracted with a mixture of phenol, chloroform, and isoamyl alcohol (25:24:1) and was precipitated by adding 1/10^th^ volume of 3M Sodium acetate and absolute alcohol to a final concentration of 70%; incubation overnight at -20°C and centrifugation at 20,000 × g for 15 min at 4 °C. The pellet was air-dried, re-suspended in 15 µl Tris acetate EDTA buffer. Samples were electrophoretically separated on a 0.8% agarose gel containing 1 µg/ml ethidium bromide and visualized under ultraviolet transillumination in a GelDoc-It Imaging System (UVP, Cambridge, UK).

### Stable transfection of U-87 MG cell line with Green Fluorescent protein tagged LC3 and autophagy assay

The cDNA encoding the microtuble-associated protein light chain 3 (MAP1LC3) was amplified from the RNA isolated from U-87 MG cells using the primers *Xho*ILC3F 5’ GATCTCGAGCCATGCCGTCGGAGAAGACCTT3’ and *Pst*ILC3R 5’ CCGTCGACTGCAGTTACACTGACAATTTCATCC3’. The PCR product was purified and cloned between the *Xho*I and *Pst*I sites of pEGFP-C1 (Clontech) and positive clones were sequenced completely. U-87 MG cells seeded in six well plates were transfected with the generated plasmid pEGFP-LC3 using lipofectamine 2000 (Invitrogen) as per manufacturer’s protocols. After 24h of transfection, cells were selected by addition of G418 at a concentration of 1mg/ml for 2 weeks. Cells were trypzinised to single cells and washed with PBS and suspended in DMEM with 20% FBS. Cell sorting was done by using FACS ARIA Flow Cytometer with sorter (BD Biosciences) for 10000 events for GFP (+) expressing cells. GFP expressing cells were further expanded in selection medium containing G418 and the sorting was repeated twice to generate the stable cells. Transiently GFP alone expressing U-87 MG cells and stable GFP-LC3 expressing U-87 MG cells were infected with CHIKV at a MOI 1 for 24h. Uninfected cells were kept as control. The cells were fixed with 4% paraformaldehyde. Imaging was done on the fixed cells using an inverted fluorescent microscope (Nikon Eclipse Ti-S).

### Detection of XBP1 mRNA splicing

U-87 MG cells were infected with the virus and the total RNA was isolated from the cells at the end of the required incubation time by Trizol method as per manufacturer’s protocols. The RNA samples were quantified in a NanoDrop 2000 (Thermo Scientific) spectrophotometer and treated with RQ1 RNase Free DNase (Promega) for 30 minutes at 37°C. 2µg of the total RNA was reverse-transcribed using Avian Myeloblastosis virus-reverse transcriptase (AMV-RT; Promega) for 1h at 42°C as per manufacturer’s protocol using oligo dT primers. The XBP1 cDNA was amplified using specific primers [23] (Table S1) from 1µL cDNA in a 10µl PCR reaction with GoTaq polymerase (Promega). PCR was done with an initial denaturation of 94°C for 2min; followed by denaturation at 94°C for 30s, primer annealing at 60°C for 30s, extension at 72°C for 1min, for 35 cycles. Equal volume of the amplified products were analyzed in 2% agarose gels and visualized by ethidium bromide staining. β-actin specific cDNA amplified from the samples were used as the PCR as well as the loading controls.

### Immunoblot analysis

Sodium dodecyl sulphate-polyacrylamide gel electrophoresis (SDS-PAGE) immunoblot were performed as follows. Cells were harvested with a scraper and re-suspended in phospho lysis buffer (1% NP-40, 10% Glycerol, 137mM NaCl, 20mM Tris-HCl, 20mM NaF, 1mM Na _4_P_2_O_7_, 1mM Na _3_VO_4,_ 1% Triton X-100 and protease inhibitor cocktail, all from Sigma). Protein extracts were mixed with one volume of the loading buffer according to Laemmli’s protocol. About 50-100µg of each sample was loaded onto a 10% SDS-PAGE. After the electrophoresis, proteins were electrotransferred onto a polyvinylidene difluoride membrane. Membranes were blocked in TBS containing 5% BSA for 1 h at room temperature. Membranes were incubated with primary antibodies overnight at 4°C followed by horseradish peroxidase-conjugated secondary antibody incubation for 1h at room temperature. Primary antibodies used included anti-eIF2α rabbit polyclonal antibody (CST#9722) and anti-phospho eIF2α (Ser51) rabbit polyclonal antibody (CST#9721) from Cell Signaling Technology; anti-RIG-I mouse monoclonal antibody (ALX-804-849) from Alexis Biochemicals; anti-MAVS (sc-68926), anti-TRAF6 (sc-7221), anti-TRAF3 (sc-948), anti-IκB-α (sc-371), anti-pIκB-α (sc-101713), anti-IKK α (sc-7218) from Santa Cruz Biotechnolgy Inc. ; anti-NF-κB-p65 (ab16502) from abcam^®^; anti-PARP (CST#95420 from Cell Signalling Technology; anti-β-actin mouse monoclonal antibody (A5441) from Sigma and an in-house anti-CHIKVE2 rabbit polyclonal serum. As secondary antibodies, monoclonal anti-rabbit IgG clone RG-96 (A1949, Sigma) and goat anti-mouse IgG (A9917, Sigma), were used for the respective primary antibodies. Protein bands were detected using ECL Plus^TM^ Western Blotting detection reagent (GE Healthcare, UK), and densitometric analysis was performed within a linear range in the Vision works software in a GelDoc-It Imaging System (UVP, Cambridge, UK.) The density of each band was normalized against that of the corresponding β-actin prior to calculation of the fold-change in expression levels with respect to the controls.

### Semi-quantitative RT-PCR

Complementary DNAs (cDNAs) were prepared from the infected and uninfected cells as described above and used for quantitative analysis of the pro-inflammatory mediators/cytokines mRNA expression. 1µL of the cDNA was used in a 10µl PCR reaction with GoTaq polymerase (Promega). PCR was done with an initial denaturation of 94°C for 2min; followed by denaturation at 94°C for 30s, primer annealing at 68°C for 30s, extension at 72°C for 1 min. 30 cycles of amplification were used for the specific targets and 24 cycles for β-actin, as decided from the preliminary experiments done to determine the log-phase amplification. Equal volume of the amplified products were analyzed in 1.5% agarose gels and visualized by ethidium bromide staining. Band intensities were analyzed by densitometry using the Vision works software in a GelDoc-It Imaging System (UVP, Cambridge, UK). Primers used for the study are given in the Table S1. The housekeeping gene β-actin expression was used to establish a baseline for normalization, and fold change in the expression of the target genes were compared between infected and control samples.

### Statistics

Unpaired (two-tailed) Student’s *t* tests applied at the 95% confidence level (p< 0.05) were carried out wherever required using Prism software (version 4; GraphPad Software Inc., San Diego, Calif., USA).

## Results

### CHIKV infects and replicates well in human glioblastoma cell line, U-87 MG

**Figure 1:**
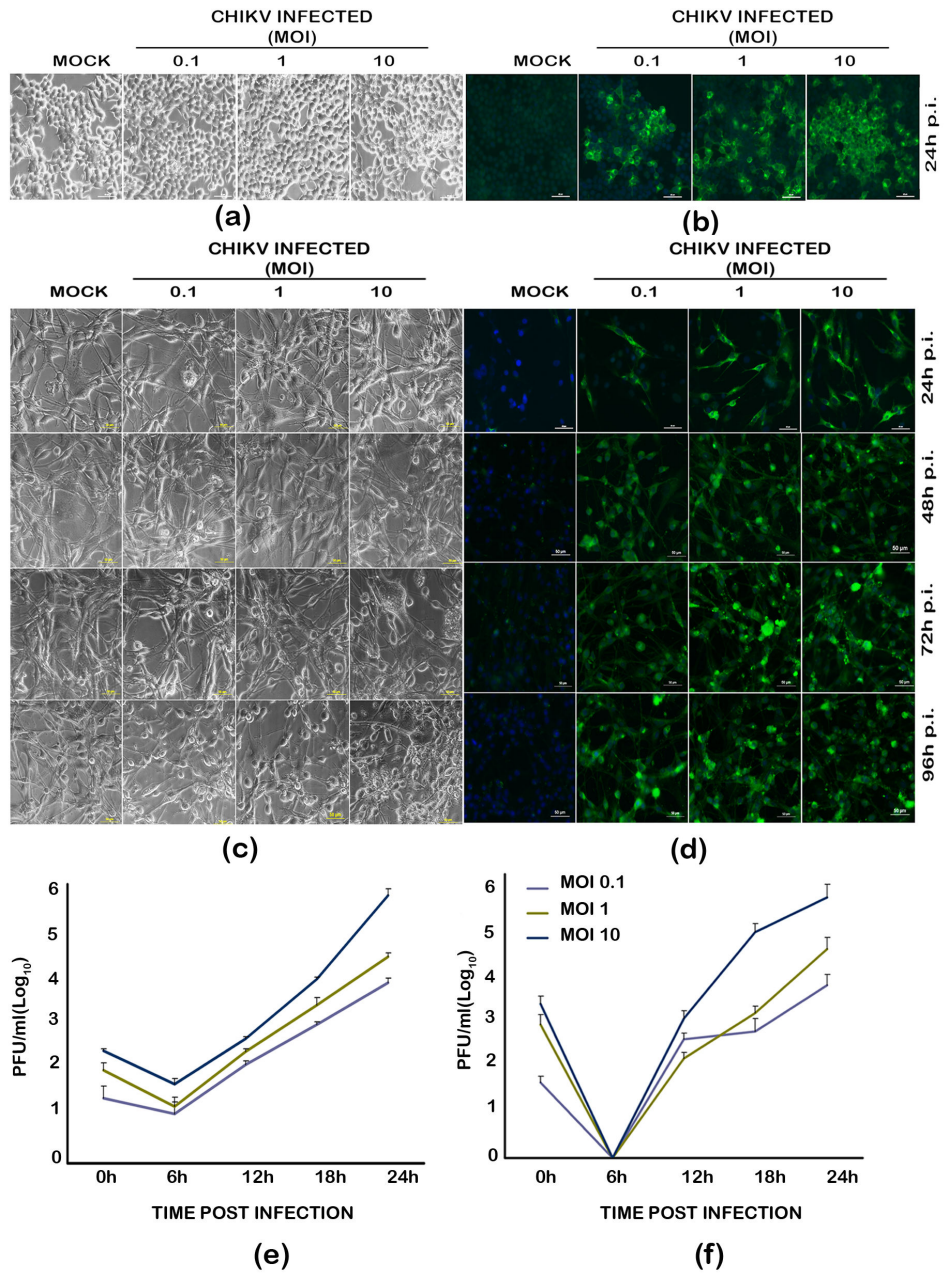
Infection of U-87 MG glioblastoma cells and HEK 293 cells with chikungunya virus. Monolayer cultures of the cells were infected at different multiplicity of infection (MOI) and observed for different duration post-infection (p.i.). Uninfected cells were kept as control. (**a**) **Bright field microscopic images of the infected HEK293 cells**. (**b**) **Immunofluorescence detection of CHIKV infection in HEK293 cells**. CHIKV infected and control cells were fixed using 4% paraformaldehyde and subjected to immunofluorescence analysis as described in methods section. The virus infection was detected using an in-house anti-CHIKV E1 envelope protein rabbit polyclonal serum at 1:10 dilution. FITC conjugated anti-rabbit IgG was used as the secondary antibody. The presence of infection is indicated by the green fluorescence foci in the infected cells. A secondary antibody control did not show any background staining at the dilutions used in the experiment (not shown). (**c**) **Bright field microscopic images showing the cytopathic changes in the infected U-87 MG cells**. Changes such as rounding and intracytoplasmic granulation are visible at 72h p.i, but they are much more pronounced at 96h p.i. The control cells remain intact and healthy at 96h p.i. Scale bar represents 50µm. (**d**) **Immunofluorescence detection of CHIKV infection in U-87 MG cells**. (**e**) **& (f) Kinetics of virus release in the culture supernatants from CHIKV infected HEK293 cells (e) and U-87 MG cells (f)**. Culture supernatants were collected at various time points post-infection and the virus plaque forming units were detected separately in confluent monolayer cultures of Vero cells as described in the methods. The values indicate the average of the number of plaques from three independent experiments, each in turn calculated from three dilutions of the sample giving <100 plaques, and represented as log_10_ values.

In order to understand the susceptibility of U-87 MG to CHIKV infection, the cells were infected with RGCB355/KL08 strain at three different multiplicities of infection (MOI 0.1, 1, and 10) and were observed under a microscope at 24h, 48h, 72h and 96h post infection (p.i.). In parallel, HEK293 cells, already known to be susceptible for CHIKV infection (Table 1), were also used for infection. Both U-87 MG and HEK293 cells appeared apparently healthy at 24h p.i (Figure 1a & c). However, the U-87 MG cells started showing signs of infection subsequently by visible cytopathic effect (CPE) in a dose and time dependent manner (Figure 1c). The infection led to rounding up of cells, granulation and disintegration of cell body from the dendritic projections, eventually leading to the detachment of the cells, cell lysis and death. Cytopathic effects were not visible in infected HEK293 cells at these time points (Data not shown). In both cell lines, the virus replication could be detected by immunofluorescence for viral envelope protein expression as distinct cytoplasmic fluorescence from 24h p.i. (Figure 1b & d). In infected U-87 MG cells, wherein multiple time points were tested, the envelope protein expression level increased depending on the duration post-infection (Figure 1d). In plaque assays of the culture supernatant from infected U-87 MG cells and HEK293 cells to study the kinetics of infection, progeny virions could be detected as early as 12h p.i. (Figure 1e & f). The virus concentration in the culture supernatants reached up to approximately 10^3^ to 10^6^ plaque forming unit/ ml at 24h in proportion with the starting MOI.

### CHIKV induces apoptosis in infected U-87 MG cells

**Figure 2:**
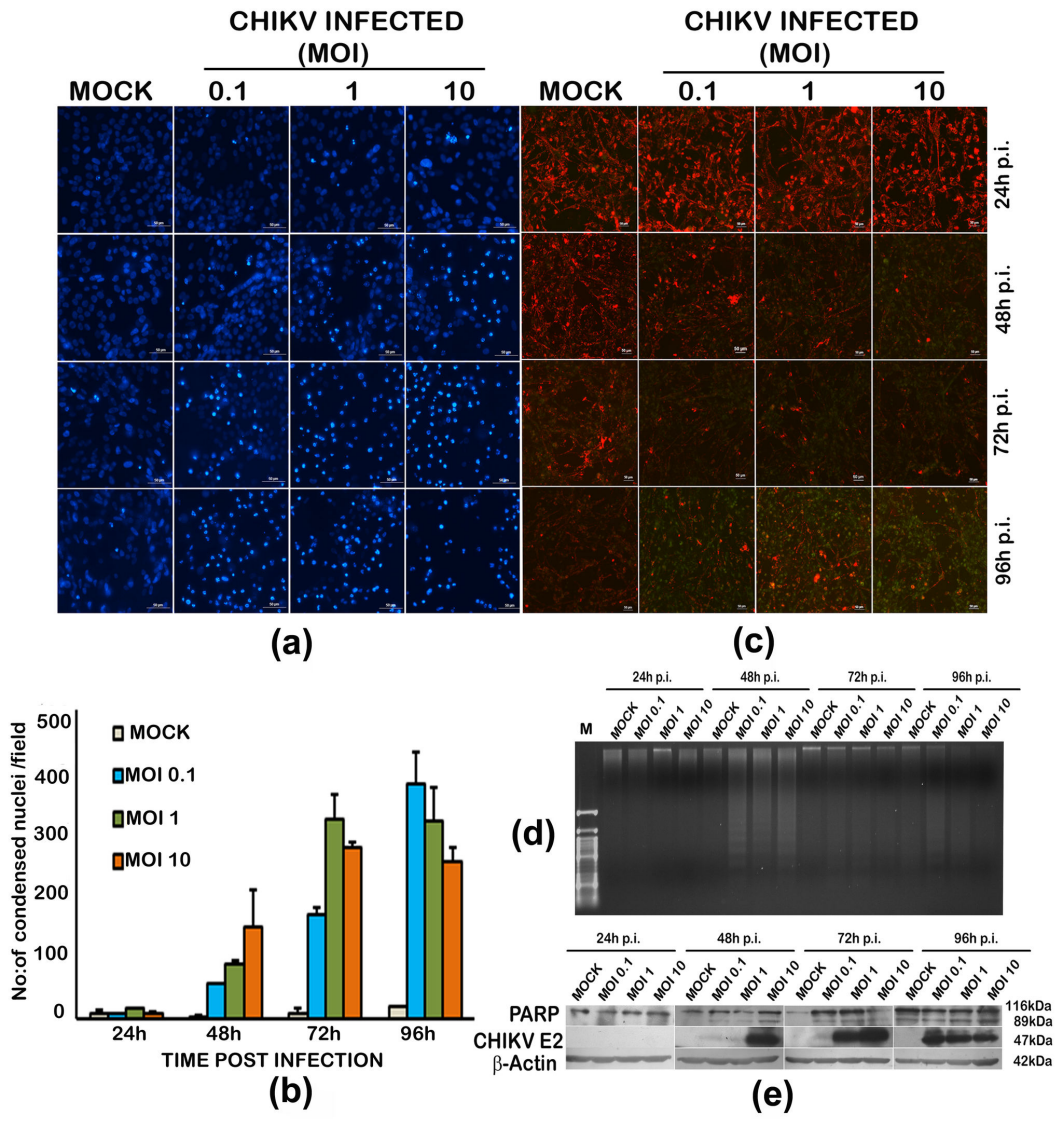
Induction of apoptosis by chikungunya virus in U-87 MG cells. (**a**) **Evidence of nuclear condensation in infected cells upon Hoechst staining**. Monolayer cultures of the cells were infected at different multiplicity of infection (MOI) and observed for different duration post-infection (p.i.). Uninfected cells were kept as control. Cells were stained with nuclear staining dye Hoechst 33342 as described in methods section and viewed under a fluorescent microscope. Scale bar represent 50µm. (**b**) **Quantitative estimation of the condensed nuclei in infected cells**. Each value is an average of four fields from three independent experiments. (**c**) **Mitochondrial membrane potential loss in infected cells**. Cells were stained with JC1 dye and the membrane potential loss is seen as loss of red fluorescence and increase in green fluorescence pronounced at 96h p.i. (**d**) **DNA** **fragmentation** **analysis** **by** **Agarose** **gel** **electrophoresis**. Total genomic DNA (1µg) isolated from CHIKV infected and uninfected cells were subjected to electrophoresis in a 0.8% agrose gel and visualized by ethidium bromide staining in a UV transilluminator. DNA fragmentation indicating apoptosis is visible in infected cells from 48h onwards. (**e**) **Western blot of infected and uninfected cells**. PARP-cleavage evidenced by presence of an 89kDa cleaved protein in infected cells. Detection of envelope protein expression using anti-CHIKV E2 antibody was used for confirming presence of the virus. β-actin expression serves as the loading control.

Nuclear condensation, loss of mitochondrial membrane potential, the poly (ADP-ribose) polymerase*-*1 (PARP-1) cleavage and DNA fragmentation are cardinal changes in apoptosis. In U-87 MG cells infected with CHIKV the nuclear condensation was evident as early as 24h p.i. (Figure 2a) and the number of intensely staining nuclei increased as the infection proceeded (Figure 2a & b). The intact mitochondrial membrane potential (MMP) retains the red fluorescence in JC1 stained healthy cells; whereas the transition to green fluorescence with loss of red fluorescence indicates MMP loss as apoptosis sets in. In infected cells, there was pronounced MMP loss at 96h p.i. (Figure 2c). Analysis of DNA fragmentation by agarose gel electrophoresis indicated the ladder pattern generated by the DNA cleaved at nucleosomes as a consequence of apoptosis (Figure 2d). The fragmentation was clear at 48h p.i. However, at 72h and 96h p.i, the fragments were poorly visible due to extensive cleavage by increased apoptotic response. Activation of caspases during apoptosis leads to the cleavage of PARP-1. In infected cells, the cleaved fragments of PARP-1, along with the mother band could be detected from 48h p.i in a dose-dependent manner and the cleavage coincided with the detectable expression of E2 envelope glycoprotein of CHIKV in the western blot (Figure 2e). In mock infected cells also there was a detectable PARP-1 cleavage at 96h p.i. consistently present in the multiple experiments we carried out, indicating a mild degree of apoptosis which could be attributed to low serum (2%) in the medium in which the cells were grown post-infection.

### CHIKV induces autophagy and endoplasmic reticulum stress in infected U-87 MG cells

**Figure 3:**
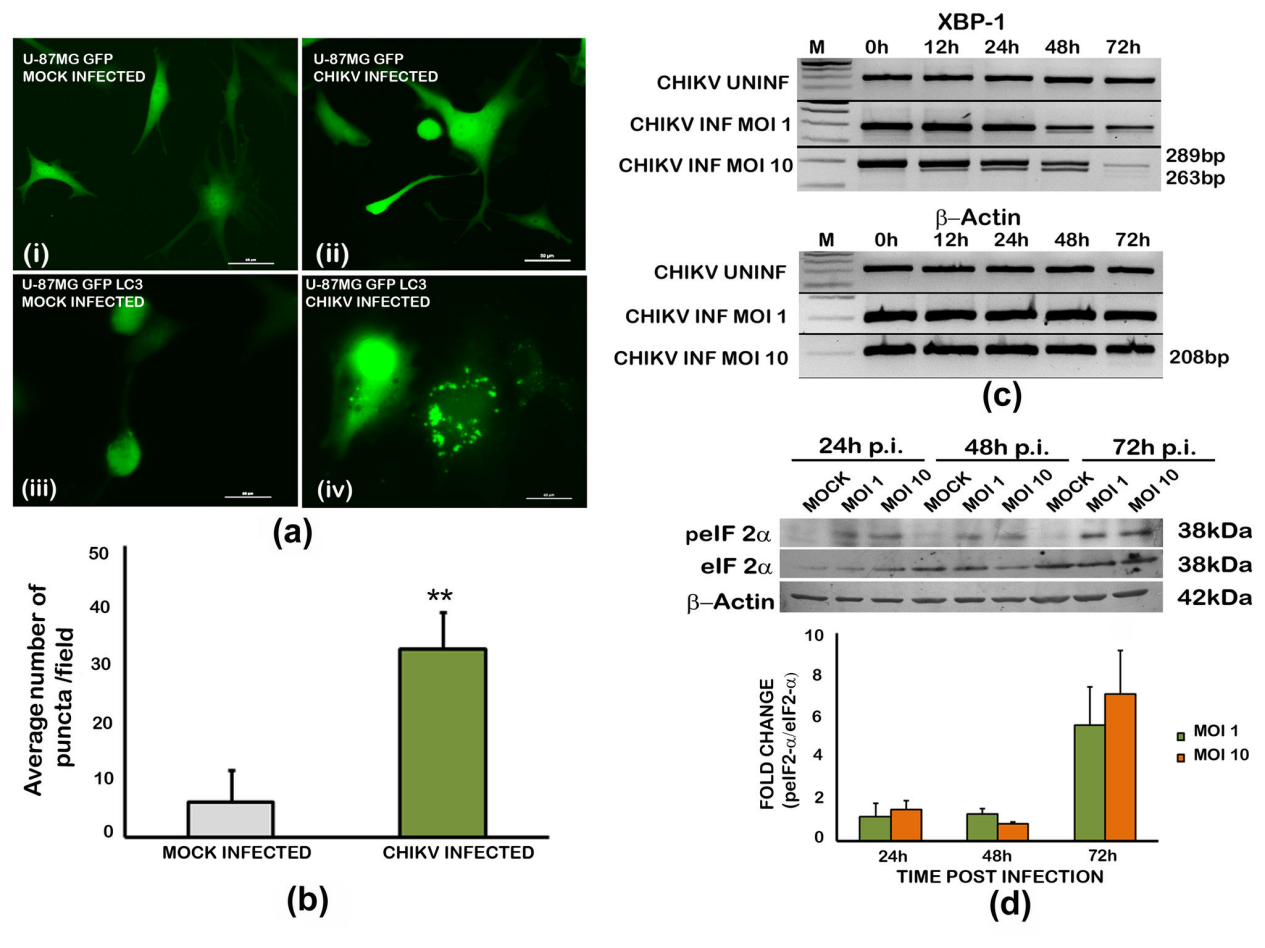
Induction of autophagy and endoplasmic reticulum stress in chikungunya virus infected U-87 MG cells. (**a**) **Punctae formation indicating the autophagosomes**. U-87 MG cells stably transfected with a GFP-tagged light chain 3 (LC3) expression-construct, and cells transiently transfected with GFP expressing plasmid alone were infected with CHIKV, and observed 24h post-infection. Distinct punctae formation by LC3 recruitment to autophagosomes is visible in the infected cells expressing GFP-LC3. (**b**) **Quantitation of the autophagy response**. The average number of puncta/field in infected and uninfected cells is given. Values are Mean ±SD from four different microscopic fields and two independent experiments. ‘**’ indicates significance level p<0.005 (**c**) **XBP-1** **mRNA** **splicing** **indicating** **ER** **stress** **in** **infected** **cells**. Total RNA was isolated from infected and uninfected cells at different time points and subjected to reverse-transcription PCR and agarose gel electrophoresis as described in the methods. The splice variant of XBP-1 (263bp) along with the original mRNA (289bp) is detectable in CHIKV infected cells from as early as 12h post-infection, but is more pronounced at 72h post infection in cells infected at an MOI of 10.Amplification of β-actin mRNA serves as the loading control. (**d**) **eIF2α phosphorylation in CHIKV infected U-87 MG cells**. Total cell lysate from the infected and uninfected cells was isolated 24h, 48 and 72h p.i. 50µg of total protein was subjected to SDS-PAGE and western blot as described in the methods using anti- eIF2α and anti-phopho-eIF2α antibodies. The band intensities of phopho-eIF2α and eIF2α expression, determined from densitometric analysis, were initially normalised independently to that of corresponding β-Actin, and the ratio of phopho- eIF2α to eIF2α expression was calculated. The fold change in expression was calculated relative to the protein expression in uninfected controls at the respective time points. Values are Mean ± SD from three independent experiments.

The induction of autophagy in U-87 MG cells was studied using a stable cell line expressing the cytoplasmic autophagic marker, microtuble-associated protein light chain 3 (LC3). LC3 is conjugated to phosphatidylethanolamine during autophagy and is targeted to autophagic membranes which can be visualized as distinct punctae by microscopy. In our experiments, LC3 tagged to GFP (LC3-GFP) formed clear distinct punctae in infected cells (Figure 3a) indicating the formation of autophagosome. The number of punctae in infected cells at 24h p.i was significantly higher than that in the uninfected cells (Figure 3b) indicating that it correlates with infection. There was no punctae formation detectable in infected cells transfected with GFP plasmid alone, or in uninfected controls.

ER stress leads to an unconventional splicing of the transcripts for X-box binding protein-1 (XBP1) mRNA resulting in a 26bp-difference spliced products. The activation of ER stress in the U-87 MG cells was analysed by XBP1 mRNA splice analysis at MOI 1 and MOI 10. In CHIKV infected samples-a spliced XBP1 (sXBP1; 263bp) and un-spliced XBP1 (uXBP1; 289bp) were obtained whereas in mock infected only uXBP1 was observed (Figure 3c). In CHIKV infection at MOI 1 there was no splicing till 24hr post infection and at later time points splicing occurs. At MOI 10 the onset of XBP1 splicing was observed even at 12hr post infection onwards. Increased rate of XBP1 splicing was observed in 24 hr, 48 hr and 72 hrs post infection.

During ER stress, the shutting off of the global protein synthesis is regulated in part through enhancing the phosphorylation level of the eukaryotic initiation factor eIF2α, which in turn regulates the mRNA translation. In CHIKV infected U-87 MG cells, enhanced phosphorylation of the eIF2α was observed at MOIs 1 and 10 (Figure 3d). The fold-change in the level of phosphorylated eIF2α with respect to un-infected control was significant at all time points and in both MOI, and varied from two fold to seven fold. This further supported the occurrence of ER stress subsequent to infection.

### CHIKV augments pro-inflammatory cytokine response in infected U-87 MG cells

**Figure 4:**
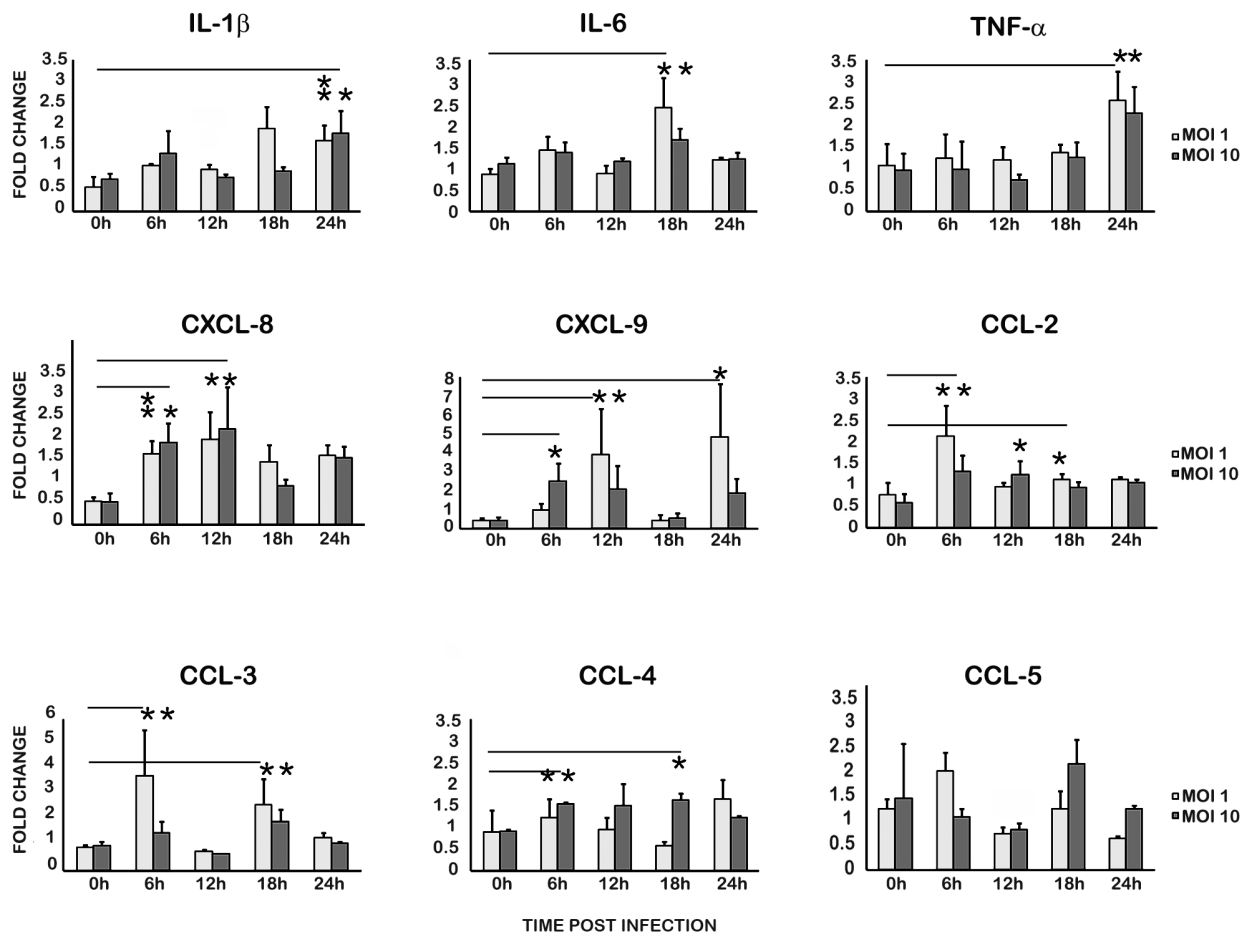
Modulation of the transcripts of pro-inflammatory cytokines in CHIKV infected U-87 MG cells. Total RNA from the infected and uninfected cells was isolated 24h p.i and was subjected to semi-quantitative RT-PCR as described in the methods. The PCR band intensities of the cytokine cDNA amplicons, determined from densitometric analysis after agarose-gel electrophoresis, were initially normalised to that of corresponding β-Actin amplicon, and the fold change in expression was calculated relative to the gene expression in uninfected controls at the respective time-points. Values are Mean ± SD from three independent biological experiments each done in duplicate. ‘*’ indicates statistically significant (p<0.05) modulation with respect to the basal level (0h) gene expression.

To understand the inflammatory cytokine response of U-87 MG cells during CHIKV infection the transcript level of 14 cytokines was analysed. These cytokines were selected based on previous reports on their perceived roles in CHIKV infection. Analyses by the semi- quantitative PCR demonstrated an increase in the mRNA expression of most of the investigated cytokines (Figure 4; Figure S1). Wide variations in the mRNA expression kinetics were observed depending on the MOI. RT-PCR amplification of IFN α, IFN β, IFN γ, IL-10, and CXCL-10 (IP 10) did not yield any amplicons from either infected or uninfected U-87 MG cells. The transcript levels of pro-inflammatory cytokines like IL-1β, IL-6 and TNF-α were found to increase from 18h p.i. CXCL-8 (IL-8) and CXCL-9 mRNAs were up-regulated in 6h p.i. Of this CXCL-9 transcript level is modulated four to five folds in cells infected with an MOI 1. The CCL members CCL-2(MCP-1), CCL-3(MIP 1α), CCL-4(MIP 1β) were found to be modulated in its transcript level while the modulation of CCL5 (RANTES) was not significant.

### Proteins of the RLR pathway family are modulated in CHIKV infected U-87 MG cells

**Figure 5:**
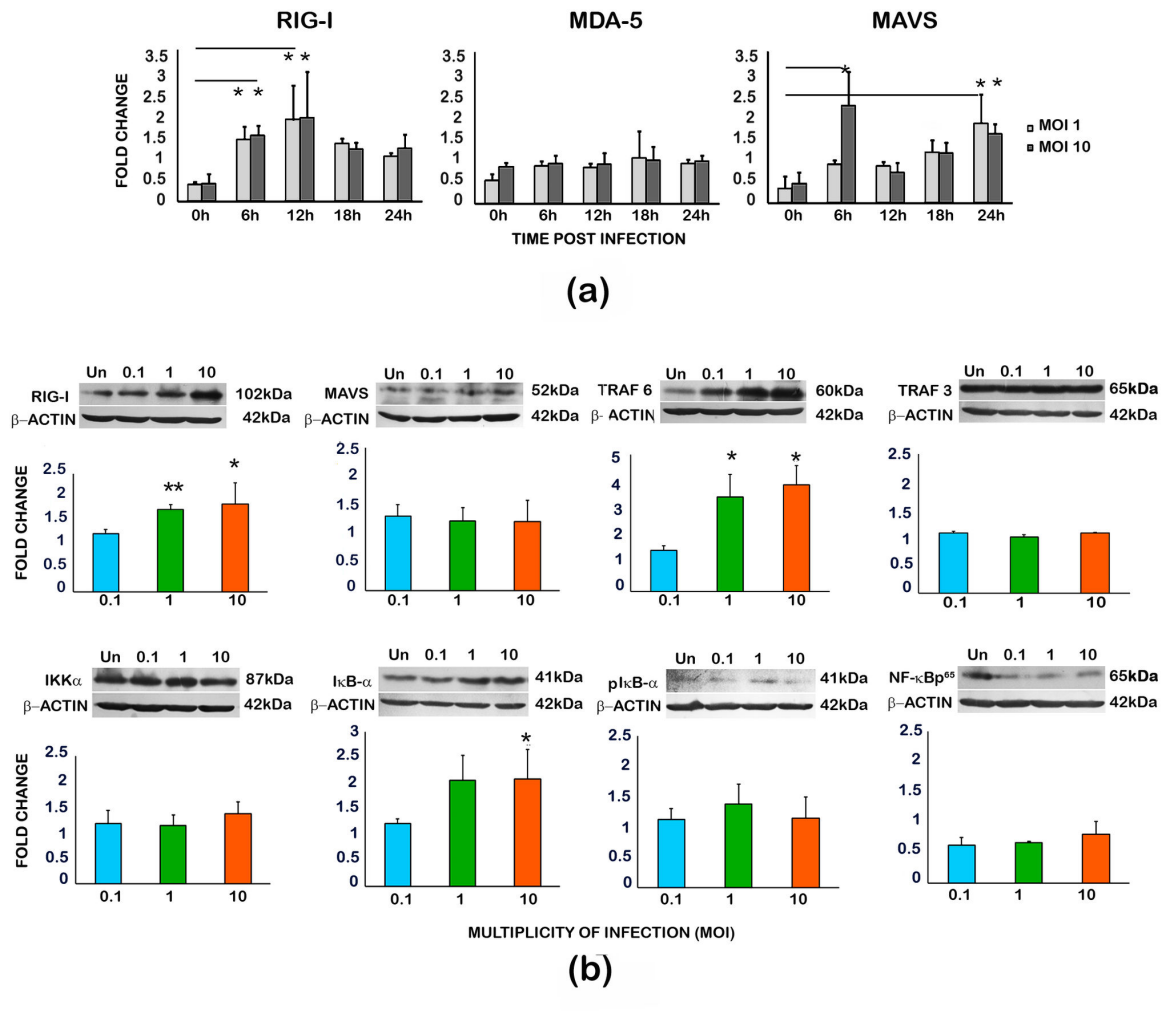
Modulation of the innate immune response sensors in CHIKV infected U-87 MG cells. **A**: **Semi-quantitative RT-PCR**. Total RNA from the infected and uninfected cells was isolated 24h p.i and was subjected to semi-quantitative RT-PCR as described in the methods. The PCR band intensities of the target cDNA amplicons, determined from densitometric analysis after agarose-gel electrophoresis, were initially normalised to that of corresponding β-Actin amplicon, and the fold change in expression was calculated relative to the gene expression in uninfected controls at the respective time-points. Values are Mean ± SD from three independent biological experiments each done in duplicate. ‘*’ indicates significant modulation with respect to the basal level (0h) gene expression. **B**. **Western blot and quantitation by densitometric analysis**. Total cell lysate from the infected and uninfected cells was isolated 24h p.i and 50-100µg of total protein was subjected to SDS-PAGE and western blot as described in the methods. The band intensities of the targets, determined from densitometric analysis, were initially normalised to that of corresponding β-Actin, and the fold change in expression was calculated relative to the protein expression in uninfected controls at 24h p.i. Values are Mean ± SD from three independent experiments. ‘*’ indicates statistically significant (p<0.05) modulation with respect to the expression in the uninfected cells.

Role of the cytosolic sensors of the RLR family was checked in CHIKV infected U-87 MG cells. Upon the transcript level analysis of the members in RLR family, up-regulation of RIG-I was evident within 6h of infection along with the up-regulation of its adaptor protein MAVS, where as there was no significant modulation in MDA 5 expression (Figure 5a). At the protein level, the RIG-I expression was 1.8 fold up-regulated at 24 h and a clear difference in the expression level of RIG-I was observed according to the viral dosage (Figure 5b). There was only a slight increase in MAVS expression. Among the TRAF family members that play a critical role in RIG-I signalling, TRAF6 significantly up-regulated (3.5 fold) at both MOI 1 and 10 indicating their role in pro-inflammatory cytokine secretion, where as there was no modulation in TRAF3. The IκBα was up-regulated where as the molecules IKKα and NF-κB p65 did not show significant changes at protein level.

## Discussion

In our study, the interferon-response deficient U-87 MG cells were found to get readily infected by the CHIKV strain RGCB355/KL08 and develop visible cytopathic effects (CPE) as in Vero cells, another IFN-response deficient cell line of primate kidney origin [24,25]. The infection kinetics were similar to that in HEK293 cells, a non-CNS cell line which has been extensively used for CHIKV infection studies previously (Figure 1e & f; Table 1). However, no visible cytopathic changes were observed in HEK293 cells infected with this virus strain, as against some of the previous studies (Table 1).

The major mechanism of cell death induced by viruses is apoptosis, which is considered as a type of ancestral defence mechanism [26]. Several alphavirus, including Semliki Forest Virus (SFV), Sindbis virus (SINV), Venezuelan equine encephalitis virus and Ross River virus trigger apoptosis in infected mammalian cells [27]. CHIKV induced apoptosis has been described in various cell lines previously (Table 1). We could also observe elucidation of apoptosis by CHIKV in the infected U-87 MG cells indicated by DNA fragmentation, nuclear condensation and MMP loss (Figure 2a-c). A recent study suggests that CHIKV-induced apoptosis is elicited through an early caspase-9-dependent intrinsic mitochondrial pathway followed by an extrinsic caspase-8-dependent pathway [28]. Even though we did not check the activation of caspases in the present study, cleavage of PARP-1 by activated caspases, which is considered to be a hallmark of apoptosis [29], was observed in the infected cells (Figure 2d).

Cell death by apoptosis becomes more damaging to the host when cells of the CNS, especially neurons that have no regenerating capacity, are destroyed subsequent to infection. Two major cellular processes, the endoplasmic reticulum (ER) stress leading to unfolded protein response (UPR) and the autophagy, could act as the mechanisms of neuroprotection by preventing cell damage and death by the over-accumulating toxic proteins in CNS viral infections [30,31]. Several arboviruses, such as Dengue virus [32], West Nile virus [33], Semliki forest virus [27], and Japanese encephalitis virus [34], induce ER stress, evidenced by the unconventional splicing of the transcripts for X-box binding protein 1 (XBP1) mRNA, a key marker for the UPR mediated ER stress [35]. In our experiments, CHIKV infection of U-87 MG cells resulted in XBP-1splicing and an increased phosphorylation of eIF2α, and supported the earlier observations of ER stress in non-CNS cells infected by CHIKV [36]. Several positive stranded RNA viruses are known to induce autophagy [37]. Interestingly in CHIKV infection, the autophagy response has been shown to delay the cell death in HeLa cells [38] and to enhance viral replication in HEK293 cells [39]. We could observe an autophagy response in infected U-87 MG cells at 24h p.i., in agreement with these observations.

Pro-inflammatory cytokines enhance inflammation in CNS [40]. Apart from this, they are thought to play role in breaking down the endothelial barrier facilitating further viral entry into brain parenchyma and also in the infiltration of leukocytes into the brain microenvironment [12,41,42]. It was previously reported in Sindbis virus-induced encephalitis that the infected astrocytes in the brain may be the source of pro-inflammatory cytokines such as IL-6 and TNF-α [18]. In the present study the mRNAs of the CXCL family members, CXCL-8 and CXCL-9, and those of the classical pro-inflammatory cytokines like IL-1β, IL-6 and TNF-α were found to be up-regulated in CHIKV infected U-87 MG cells, though the kinetics of mRNA expression were different for each of the cytokines (Figure 4). The CXCL-9 and CXCL-10 chemokines are considered to be the contributors of persistent immune activation in CHIKV infection [43]. However, we could not detect the expression CXCL10 in infected U-87 MG cells. A curious observation was the inverse correlation of the expression levels of the transcripts of IL-6, TNF-α, CXCL9, CCL2 and CCL3 and the virus infection dose. The cells infected at a higher MOI of 10 showed a lower transcript level of these cytokines at certain time points compared to the cells infected at a lower MOI of 1. A plausible explanation could be the altered expression kinetics of these cytokine transcripts at higher MOI. More number of cells gets simultaneously infected in higher MOIs, and in these cells the cellular protein synthesis gets modified for virus replication rather than for cytokine synthesis. On the other hand, in low MOI there are more numbers of uninfected, by-stander cells present, which may respond to the paracrine effects of the early cytokines secreted from the infected cells. This could get reflected in the level of mRNA transcript in short-duration experiments. Further, a longer duration kinetic analysis and also, a protein based study, such as ELISA (which will represent the accumulated cytokines overtime, rather than transient mRNA expression levels) are needed to further validate these observations. In general, these cytokine/chemokine dysregulation and activation of glial cells may be some of the key factors for CHIKV induced neurological manifestations in clinical cases.

The cytoplasmic sensors of the Toll-like receptor (TLR) and RIG-I-like receptors (RLR)-signalling pathways of the cellular innate immune response recognise the viral RNA and induce pro-inflammatory cytokines in several CNS viral infections [44]. The involvement of (RLR) pathway in inflammatory cytokine production has been reported earlier in a few virus infection models such as astrocyte infection by vesicular stomatitis virus, neuronal infection by Japanese encephalitis virus, hepatic cell infection by HCV and endothelial cells infection by Dengue virus [45,46,47,48]. In CHIKV infected fibroblasts, the activation of RLR signalling pathway has been identified [49]. On similar lines, in our study with U-87 MG cells, the activation of RLR pathway by the enhanced expression of RIG-I both at the mRNA and protein level was observed (Figure 5a& b). However, we could not find the role of MDA5, another sensor molecule in the pathway, in the inflammatory cytokine response (Figure 5a). The role of mitochondrial antiviral signalling (MAVS /IPS-I), an adapter molecule downstream in the pathway with which RIG-I and MDA5 interacts, has been shown previously in CHIKV infection [49,50]. Subsequently in the pathway, the activated MAVS dimerizes and binds with the tumour necrosis factor (TNF) receptor-associated factors (TRAFs)-TRAF2 and TRAF6-triggering the pro-inflammatory signalling pathway, and TRAF3, stimulating the IFN-response [51]. In our analysis of the CHIKV-infected U-87 MG cells done at 24h p.i., a significant up-regulation of TRAF6 protein, rather than TRAF3, was observed. The TRAF6, an E3 ubiquitin ligase, is a key intermediate in RLR signalling mechanism that leads to NF-κB activation and inflammatory response. In previous studies, importance of TRAF-6 activation has been shown in infection by various RIG-I sensed RNA viruses such as vesicular stomatitis virus, Newcastle disease virus, encephalomyocarditis virus, Sendai virus and also in respiratory syncytial virus [51,52], but not in CHIKV infection.

In conclusion, we find that the interferon-response deficient cell line U-87 MG is susceptible to CHIKV infection and exhibits many intracellular changes as reported for other cells that have an intact IFN system (Table 1) [53-69]. We presume that studies using this *in vitro* cellular model of CNS origin may provide observations that corroborate with the cellular responses happening in very young and very old patients during natural CHIKV CNS infection. Further, the identification and characterization of the specific virus-encoded proteins modulating these responses, and the effect of amino acid changes observed in these proteins in field isolates would define the neurovirulence of CHIKV more precisely. Also, albeit on a different perspective, apoptosis induction by CHIKV in glioblastoma cells is exciting. Glioblastoma is a highly aggressive and chemotherapy resistant malignancy which commonly has defective Type I Interferon response [21]. Our results point towards the possibility of further exploration of the CHIKV infection system using adapted or modified viruses, for developing oncolytic virotherapy approaches against this malignancy, as envisaged in earlier studies [70].

## Supporting Information

Figure S1 **Agarose gel electrophoresis of RT-PCR amplified products A: Cytokine mRNA B: Innate immune response mediators.** A representative gel of a total of six runs (three independent experiments each in duplicate).
(TIFF)

Table S1 **List of primers used in the study.**
(DOC)
