# Supplementary figures and images for: Induction of Cytopathogenicity in Human Glioblastoma Cells by Chikungunya Virus
Source: PLoS One. 2013 Sep 25;8(9):e75854. doi: 10.1371/journal.pone.0075854 (PMC3783433; doi:10.1371/journal.pone.0075854)

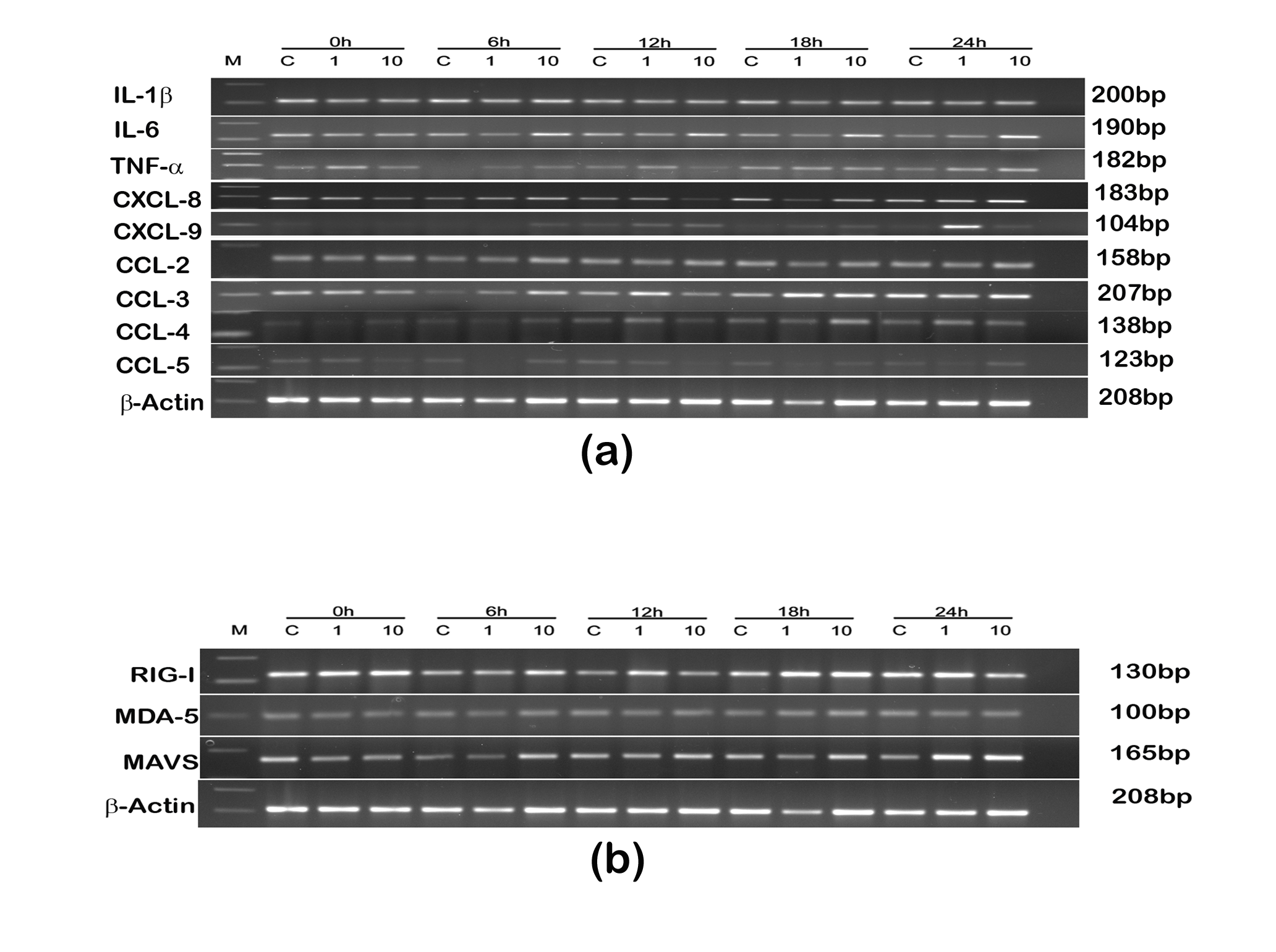

Supplement: Figure S1 — Agarose gel electrophoresis of RT-PCR amplified products A: Cytokine mRNA B: Innate immune response mediators. A representative gel of a total of six runs (three independent experiments each in duplicate). (TIFF) [file pone.0075854.s001.tiff]
