# Supplementary material for: Induction of Cytopathogenicity in Human Glioblastoma Cells by Chikungunya Virus
Source: PLoS One. 2013 Sep 25;8(9):e75854. doi: 10.1371/journal.pone.0075854 (PMC3783433; doi:10.1371/journal.pone.0075854)
Supplement: Table S1 — List of primers used in the study. (DOC) [file pone.0075854.s002.doc]

| **Name** | **Sequence (5’-3’)** | **Amplicon size** |
| --- | --- | --- |
| hXBP1F | TTACGAGAGAAAACTCATGGCC | 289bp , 263bp |
| hXBP1R | GGGTCCAAGTTGTCCAGAATGC |
| hIL-1βNF | CACGCTCCGGGACTCACAGC | 200bp |
| hIL-1βNR | GGAGAACACCACTTGTTGCTCCA |
| hIL-6NF | TCCACAAGCGCCTTCGGTCCA | 190bp |
| hIL-6NR | TGTCTGTGTGGGGCGGCTACA |
| hIL-10F | AGGCAACCTGCCTAACATGCTTCG | 200bp |
| hIL-10R | GGTTCTCAGCTTGGGGCATCAC |
| hTNF-αF | AGGCGGTGCTTGTTCCTCAGCCTC | 182bp |
| hTNF-αR | GGCTTGTCACTCGGGGTTCGA |
| hCXCL-8F | GGTGCAGTTTTGCCAAGGAGTGCT | 183bp |
| hCXCL-8R | TTCCTTGGGGTCCAGACAGAGC |
| hCXCL-9F | TGAGAAAGGGTCGCTGTTCCTGCATCA | 104bp |
| hCXCL-9R | TTTCTCGCAGGAAGGGCTTGGGGCAAAT |
| hCXCL-10F | GAACTGTACGCTGTACCTGCATCAGCA | 108bp |
| hCXCL-10R | TCTCAACACGTGGACAAAATTGGCTTGC |
| hCCL-2F | AATGCCCCAGTCACCTGCTGT | 158bp |
| hCCL-2R | TTCTGCTTGGGGTCAGCACAGA |
| hCCL-3F | TGCACCATGGCTCTCTGCAACCAG | 207bp |
| hCCL-3R | GACCCACTCCTCACTGGGGTCAGCA |
| hCCL-4F | AGTAGCTGCCTTCTGCTCTCCAG | 138bp |
| hCCL-4R | GAGCAGAGGCTGCTGGTCTC |
| hCCL-5F | TCGGACACCACACCCTGCT | 123bp |
| hCCL-5R | CTTTCGGGTGACAAAGACGACTGCT |
| hIFN-α1F | CTACGATGGCCTCGCCCTTTGC | 104bp |
| hIFN-α1R | ATCCAGGCTGTGGGTCTCAGGGA |
| hIFN-βNF | CTCCTGTGGCAATTGAATGGGAGGC | 134bp |
| hIFN-βNR | CAATGCGGCGTCCTCCTTCTGG |
| hIFN-γF | GCATCGTTTTGGGTTCTCTTGGCTG | 243bp |
| hIFN-γR | CCACACTCTTTTGGATGCTCTGGTC |
| hRIG-IF | CTCTGCAGAAAGTGCAAAGC | 130bp |
| hRIG-IR | GGCTTGGGATGTGGTCTACT |
| hMDA5F | TGGTCTCGTCACCAATGAAA | 100bp |
| hMDA5R | CTCCTGAACCACTGTGAGCA |
| hMAVS-F | GCACACTCTCAGGGAACCGGGA | 165bp |
| hMAVS-R | CCGAGGTCCGAGGCTGGTAGCTC |
| hβ-actinF | CCGCAAAGACCTGTACGCCAACAC | 208bp |
| hβ-actinR | GCTGATCCACATCTGCTGGAAGGT |

**Supplementary Table 1** Sequences of the primers used in the study
